# Five-year follow-up of OPTIC: long-term efficacy, safety, and mutation analyses of ponatinib in chronic-phase chronic myeloid leukemia from a randomized phase 2 trial

**DOI:** 10.1038/s41375-026-03009-x

**Published:** 2026-07-24

**Authors:** Hagop Kantarjian, Michael Deininger, Jane F. Apperley, Christopher Kevin Arthur, Charles Chuah, Andreas Hochhaus, Hugues de Lavallade, Jeffrey H. Lipton, Elza Lomaia, James McCloskey, Lori Maness, Michael Mauro, Beatriz Moiraghi, Carolina Pavlovsky, Gianantonio Rosti, Philippe Rousselot, Bo Chao, Alexander Vorog, L. Evan Reddick, Niti Patel, Meliessa Hennessy, Tammie Yeh, Jorge Cortes

**Affiliations:** 1 The University of Texas MD Anderson Cancer Center, Houston, TX USA; 2 University of Michigan Ann Arbor, Ann Arbor, MI USA; 3 Imperial College London, London, UK; 4 Royal North Shore Hospital, St. Leonards, NSW Australia; 5 Singapore General Hospital, Duke-NUS Medical School, Singapore, Singapore; 6 Universitätsklinikum Jena, Jena, Germany; 7 Guy’s and St Thomas’ NHS Foundation Trust, London, UK; 8 Princess Margaret Cancer Centre, Toronto, ON Canada; 9 Almazov National Medical Research Centre, St. Petersburg, Russia; 10 The John Theurer Cancer Center at Hackensack Meridian Health, Hackensack, NJ USA; 11 University of Nebraska Medical Center, Omaha, NE USA; 12 Memorial Sloan Kettering, New York, NY USA; 13 Hospital Jose Maria Ramos Mejia, Buenos Aires, Argentina; 14 Fundaleu, Buenos Aires, Argentina; 15 IRCCS Istituto Romagnolo per lo Studio dei Tumori (IRST) “Dino Amadori”, Meldola, FC Italy; 16 Centre Hospitalier de Versailles University de Versailles Saint-Quentin-en-Yvelines, Paris, France; 17 Takeda Development Center Americas Inc, Cambridge, MA USA; 18 Georgia Cancer Center at Augusta University, Augusta, GA USA

**Keywords:** Chronic lymphocytic leukaemia, Phase II trials

## Abstract

The primary analysis of the phase 2 OPTIC trial (NCT02467270) demonstrated optimal benefit:risk with response-based ponatinib dosing (45 mg once daily (QD) reduced to 15 mg QD) upon achieving ≤1% *BCR::ABL1*^IS^ in patients with tyrosine kinase inhibitor-resistant or T315I-positive chronic-phase chronic myeloid leukemia (CP-CML). Here, we report 5-year long-term outcomes. Overall, 283 patients were randomized to 45-mg, 30-mg, or 15-mg QD starting doses (*n* = 94, 95, and 94, respectively), with dose reduction to 15 mg QD upon response in the 45-mg and 30-mg cohorts. At data cutoff, 61 patients remained on trial. Median follow-up time was 75–78 months. By 5 years, 60%, 41%, and 40% of patients in the 45-mg, 30-mg, and 15-mg cohorts, respectively, achieved ≤1% *BCR::ABL1*^IS^. Five-year progression-free survival rates were 63%, 57%, and 60%, respectively, by cohort; overall survival rates exceeded 80%. In patients with a T315I mutation, 5-year rates of ≤1% *BCR::ABL1*^IS^, PFS, and OS were highest in the 45-mg cohort. Exposure-adjusted rates of adjudicated arterial occlusive events were 4.1, 3.8, and 2.0 patients per 100 patient-years, respectively, by cohort; results were comparable in T315I-positive patients. These findings support long-term clinical benefit of response-based ponatinib dosing in third-line CP-CML, especially in patients with the T315I mutation.

**Figure:**
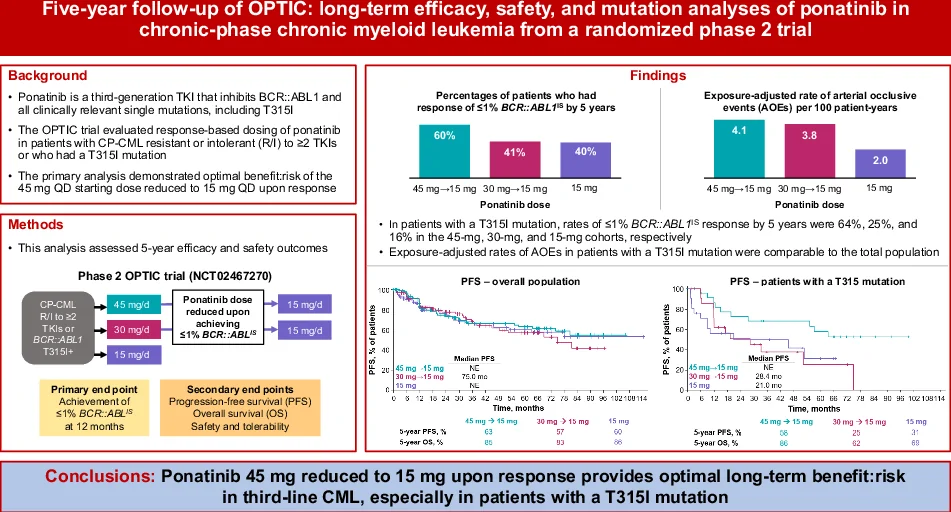

## Introduction

While chronic myeloid leukemia (CML) is now associated with long-term survival [1–3], many patients eventually require a change in BCR::ABL1 tyrosine kinase inhibitor (TKI) therapy due to development of drug resistance, often caused by emergence of *BCR::ABL1* mutations, such as the “gatekeeper” T315I mutation [4, 5]. Patients who develop resistance after multiple lines of therapy, particularly those harboring a T315I mutation, represent a population with limited treatment options and ongoing unmet clinical need.

Ponatinib is a third-generation BCR::ABL1 TKI designed to potently inhibit BCR::ABL1 regardless of mutation status, including T315I [6]. The pivotal phase 2 PACE trial demonstrated clinically meaningful and durable responses with a flat 45-mg daily dose of ponatinib in patients with chronic-phase CML (CP-CML) resistant to multiple prior TKIs [7]. However, additional follow-up of PACE and findings from the terminated phase 3 EPIC trial identified increased rates of arterial occlusive events (AOEs) [8, 9], highlighting the need to optimize dosing strategies. Pooled analyses further demonstrated a potentially dose-dependent relationship between ponatinib and AOEs [10]. These findings led to updates in prescribing recommendations and initiation of the OPTIC trial.

OPTIC was a dose-ranging phase 2 trial designed to prospectively identify the response-based dose-reduction strategy that optimizes the benefit:risk profile of ponatinib in patients with CP-CML resistant or intolerant to ≥2 prior TKIs or with a *BCR::ABL1* T315I mutation [11]. A model-based analysis of OPTIC and the primary analysis of OPTIC (primary endpoint: ≤1% *BCR::ABL1*^IS^ at 12 months; minimum follow-up: 1 year) demonstrated that a 45-mg starting dose reduced to 15 mg upon reaching a response of ≤1% *BCR::ABL1*^IS^ maximized efficacy while minimizing toxicity [11, 12].

Long-term data evaluating durability of response and mutation evolution with optimized ponatinib dosing remain essential to inform treatment decisions in later-line CP-CML, particularly for patients with baseline *BCR::ABL1* mutations such as T315I. Long-term follow-up is also critical to characterize long-term safety of ponatinib in this optimized dosing schedule.

Here, we report final, long-term efficacy, safety, and *BCR::ABL1* mutation analyses from OPTIC, with a minimum of 5 years of follow-up.

## Materials and methods

### Patients and study design

Detailed descriptions of the open-label, phase 2 OPTIC trial (NCT02467270) design and randomization procedures were previously reported in the primary publication by Cortes et al. [11]. Briefly, eligible patients were adults (age ≥18 years) with CP-CML resistant to ≥2 prior TKIs or with a *BCR::ABL1* T315I mutation in any line of therapy. *BCR::ABL1* transcript levels on the International Scale (*BCR::ABL1*^IS^) > 1% by reverse transcriptase-polymerase chain reaction (RT-PCR) were required at screening.

Patients were enrolled at 61 sites in 19 countries, spanning North America, Europe, Latin America, and the Asia-Pacific region. Patients were randomly assigned in a 1:1:1 ratio to receive oral ponatinib once daily at a starting dose of 45 mg (45-mg cohort), 30 mg (30-mg cohort), or 15 mg (15-mg cohort). Randomization was stratified by age (≥60 vs <60 years) and history of arterial hypertension, diabetes mellitus, and/or hyperlipidemia (yes/no). In the 45-mg and 30-mg cohorts, dose was reduced to 15 mg once daily upon achievement of response (≤1% *BCR::ABL1*^IS^). In all cohorts, dose reduction to a minimum of 10 mg daily was permitted for adverse events (AEs). The dose could be re-escalated to the assigned starting dose if a patient experienced confirmed loss of ≤1% *BCR::ABL1*^IS^ on 2 consecutive assessments and did not have ongoing AEs requiring dose reduction.

An end-of-treatment (EOT) visit occurred after the last dose of study drug or the decision to discontinue treatment. Upon study termination after the 5-year data cutoff (May 15, 2024), as predefined in the protocol, patients who continued to derive clinical benefit from ponatinib were transitioned to a post-trial access (PTA) program; for these patients, EOT visit corresponds to study completion and transfer to PTA.

This study was conducted in accordance with the Declaration of Helsinki. The study protocol, amendments, and informed consent form were approved by the institutional review board/ethics committee of each study site. All patients provided written informed consent.

### End points

The primary end point was achievement of ≤1% *BCR::ABL1*^IS^ at 12 months. Secondary efficacy end points included ≤0.1% *BCR::ABL1*^IS^ (MMR), ≤0.01% *BCR::ABL1*^IS^ (MR4), and ≤0.0032% *BCR::ABL1*^IS^ (MR4.5) rates, progression-free survival (PFS), and overall survival (OS).

Safety and tolerability end points included rates of AEs, including adjudicated AOEs, serious AEs, and treatment discontinuations, and dose modifications due to AEs. *BCR::ABL1* mutations were assessed at baseline and at EOT or upon transition to PTA.

### Assessments

Molecular responses were analyzed using peripheral blood samples collected at baseline, every 3 cycles (28 days/cycle) through cycle 24, and every 3 cycles thereafter for patients who had completed <24 total cycles in the study or every 6 cycles thereafter for patients who had completed ≥24 total cycles in the study, and at EOT or when patients transitioned to PTA. *BCR::ABL1*^IS^ was quantified by RT-PCR according to standard criteria [13].

Peripheral blood samples collected at baseline and EOT or PTA transfer were analyzed for *BCR::ABL1* mutations using Sanger sequencing when ≥100 transcript copies were present. All molecular and sequencing analyses were performed at a central laboratory (ICON Specialty Laboratory/MolecularMD, Portland, OR).

PFS events were defined as death, progression to accelerated phase (AP) CML (no extramedullary disease and any of the following: 15–29% blasts, ≥20% basophils, or ≥30% blasts + promyelocytes [but <30% blasts] in peripheral blood or bone marrow; <100 ×10^9^ platelets/L in peripheral blood unrelated to therapy; or cytogenic or genetic evidence of clonal evolution) or blast phase (BP) CML (≥30% blasts in peripheral blood or bone marrow or extramedullary disease other than hepatosplenomegaly), loss of complete hematologic response (CHR; in absence of cytogenetic response) confirmed in complete blood counts obtained ≥4 weeks apart, loss of major cytogenetic response by bone marrow cytogenetic assessment, or increasing white blood cell (WBC) count in patients without CHR (defined as doubling of WBC to >20,000 on 2 occasions ≥4 weeks apart after the first 4 weeks of therapy) [14]. OS was defined as the interval between the first dose of ponatinib and death from any cause.

AEs were graded according to the National Cancer Institute Common Terminology Criteria for Adverse Events, version 4.0. An independent cardiovascular endpoint adjudication committee, composed of independent experts with experience and training appropriate for reviews of cardiovascular, AOE, heart failure, and venous thromboembolic event endpoints, reviewed all documentation related to cardiovascular AEs to determine if they met charter-defined criteria for AOEs.

All analyses, except EOT *BCR::ABL1* mutation analyses, used a data cutoff date of May 15, 2024, corresponding to ≥5 years of follow-up for all remaining patients. Analyses of EOT samples used the data cutoff date of April 23, 2025, corresponding to completion of PTA transitions.

### Statistical analysis

Sample size determination and handling of missing data for OPTIC has been previously reported [11]. Molecular responses were analyzed in patients with *BCR::ABL1* e13a2/e14a2 transcripts at baseline (intention-to-treat [ITT] population). Survival outcomes were analyzed in all randomized patients. Safety and tolerability and mutation analyses were conducted in patients who received ≥1 dose of ponatinib (safety population). Molecular responses, survival outcomes, and safety and tolerability data were analyzed in all patients and stratified by baseline *BCR::ABL1* mutation status (no mutations, T315I mutation, and non-T315I mutations).

All data were summarized descriptively. Time-to-event outcomes (e.g., duration of response, PFS, OS) were analyzed using the Kaplan–Meier method. Exposure-adjusted serious treatment-emergent AE (TEAE) and AOE rates were calculated as the number of patients with the event divided by total treatment exposure time.

## Results

### Patients

**Fig. 1:**
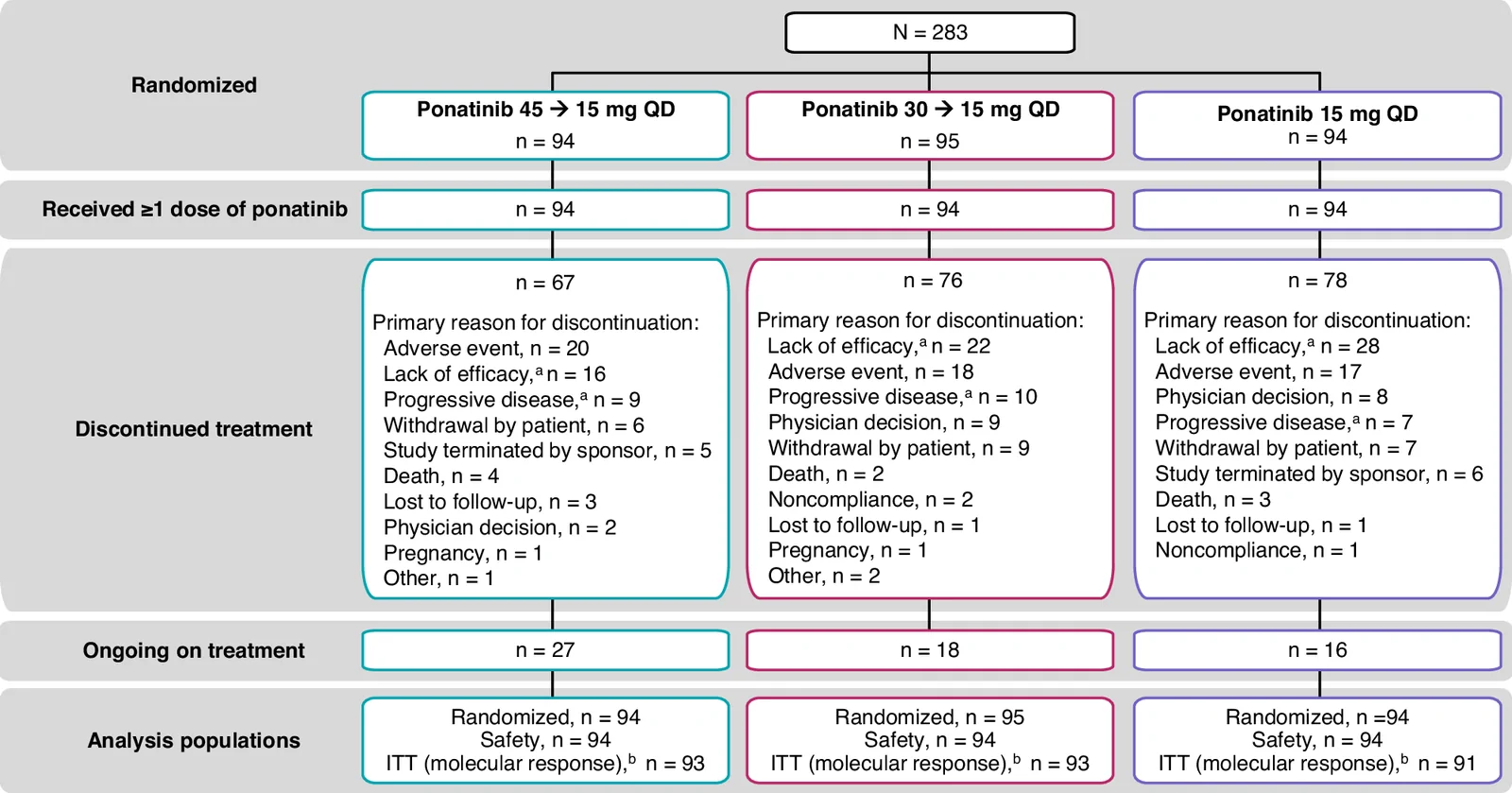
Patient disposition in the OPTIC trial as of May 15, 2024 (data cutoff date). ^a^Per protocol, lack of efficacy was defined as the absence of MCyR at 12 months, absence of CHR by 3 months, or loss of ≤1% *BCR::ABL1*^IS^ in the absence of dose re-escalation or continued loss of ≤1% *BCR::ABL1*^IS^ after 6 months following dose re-escalation; disease progression means progression of disease to AP- or BP-CML. ^b^Molecular response rates were evaluated in the ITT population, which included all patients who were randomized and for whom *BCR::ABL1*^IS^ could be measured (i.e., patients who had the e13a2/e14a2 transcript type), regardless of whether they received the assigned treatment. AP acute phase, BP blast phase, CHR complete hematologic response, CML chronic myeloid leukemia, ITT intention to treat, MCyR major cytogenetic response, QD once daily.

The OPTIC patient population has been described previously [11]. A total of 283 patients were randomly assigned to ponatinib starting doses of 45 mg (*n* = 94), 30 mg (*n* = 95), or 15 mg (*n* = 94); 282 received ≥1 dose of ponatinib. In total, 277 randomized patients were evaluable for *BCR::ABL1*^IS^ response (Fig. 1). Baseline demographics and clinical characteristics were balanced across treatment arms (Supplementary Table S1). Importantly, nearly all patients (99%) were resistant to prior TKI therapy, and 55% had received ≥3 prior TKIs, reflecting a heavily pretreated third-line population.

At baseline, 24% (67/282) had a *BCR::ABL1* T315I mutation, 17% (48/282) had *BCR::ABL1* mutations other than T315I, and 58% (163/282) had no detectable *BCR::ABL1* mutations; baseline *BCR::ABL1* mutation status was not available in 1% (4/282). Ten patients (4%) had a T315I mutation in combination with ≥1 additional non-T315I mutation.

At the 5-year data cutoff, 61 patients remained on ponatinib; a greater proportion continued treatment in the 45-mg cohort (29%, 27/94) than in the 30-mg (19%, 18/95) or 15-mg (17%, 16/94) cohorts. Median follow-up was 77.9 months in the 45-mg cohort, 75.1 months in the 30-mg cohort, and 75.4 months in the 15-mg cohort. Among the 282 patients treated with ponatinib, 85 (30.1%) remained on treatment for ≥5 years (range, 60–105 months). Across cohorts, the most common reasons for discontinuation were lack of efficacy (23%), AEs (19%), and progressive disease (9%). Discontinuation due to lack of efficacy occurred more frequently with lower starting doses (15 mg, 30%; 30 mg, 23%) compared with 45 mg (17%). Median dose intensity at data cutoff was 27.7 mg/day in the 45-mg cohort, 23.5 mg/day in the 30-mg cohort, and 14.7 mg/day in the 15-mg cohort; median dose intensity at 1 year was 15.0 mg/day, 27.9 mg/day, and 15.0 mg/day, respectively. Dose reductions as per protocol for efficacy- or AE-related reasons occurred in 44% and 48% of patients in the 45-mg cohort, 23% and 37% in the 30-mg cohort, and 0% and 32% in 15-mg cohort, respectively.

### Molecular response

**Fig. 2:**
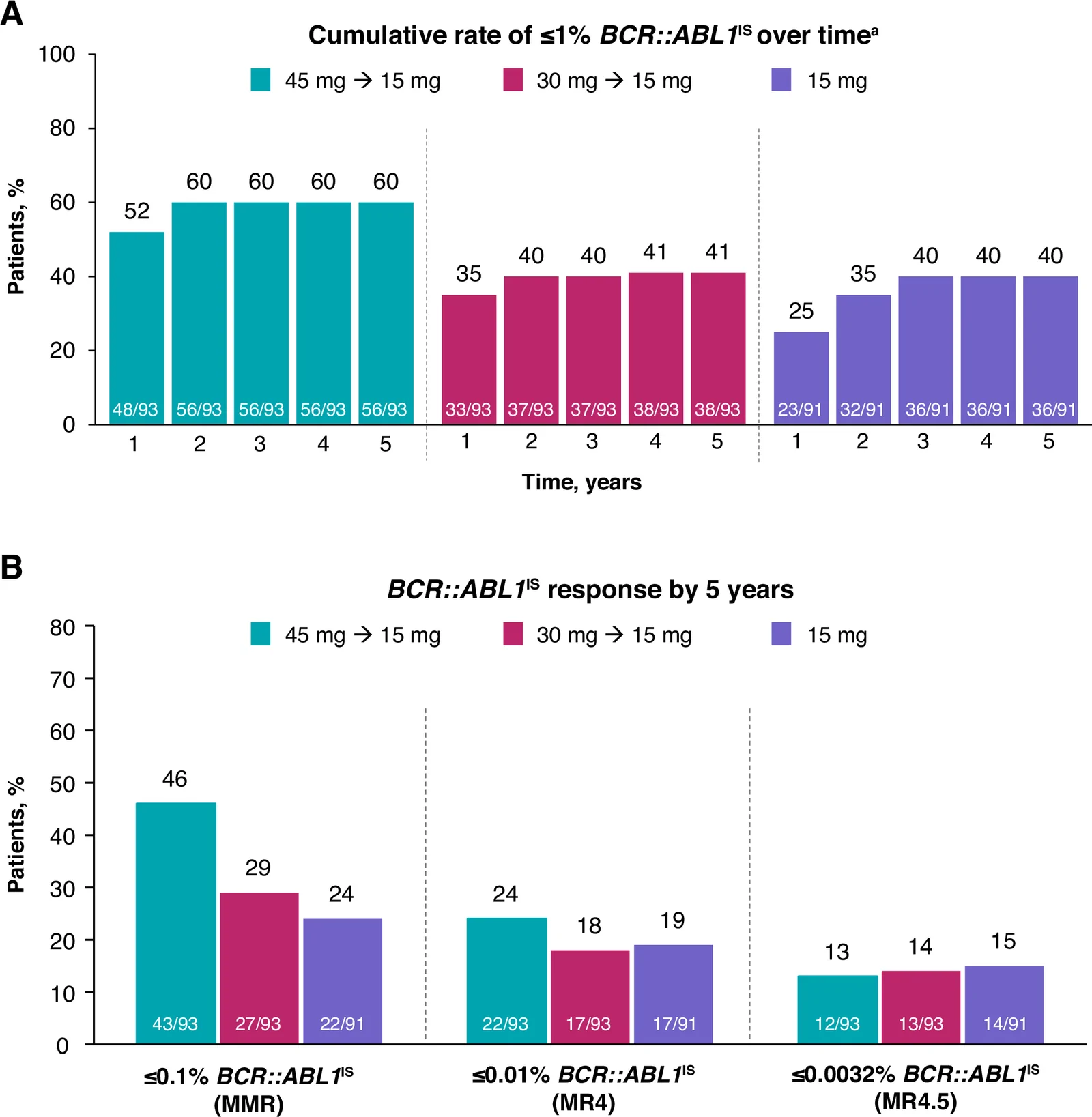
Response to ponatinib. Cumulative rates of **A** ≤1% *BCR::ABL1*^IS^ response over time and **B** MMR (≤0.1% *BCR::ABL1*^IS^), MR4 (≤0.01% *BCR::ABL1*^IS^), and MR4.5 (≤0.0032% *BCR::ABL1*^IS^) by 5 years in the ITT population. ^a^Numbers of patients with ≤1% *BCR::ABL1*^IS^ were counted on cumulative basis by each time point; a patient with response was counted only once. Responses were measured at each patient’s yearly visits. ITT intention to treat, MMR major molecular response.

In the ITT population, 60%, 41%, and 40% of patients achieved ≤1% *BCR::ABL1*^IS^ by 5 years in the 45-mg, 30-mg, and 15-mg cohorts, respectively; most responses occurred by 2 years, with no new responses after 4 years (Fig. 2A). Median time to response was 6.0 months (range, 2.9–21.1) in the 45-mg cohort, 3.1 months (range, 2.9–45.0) in the 30-mg cohort, and 6.2 months (range, 2.9–33.1) in the 15-mg cohort. Median duration of ≤1% *BCR::ABL1*^IS^ response (i.e., time to first loss of response) was not reached in any cohort; by Kaplan–Meier estimation, the proportion of patients maintaining the response at 5 years was 68.8% (95% confidence interval [CI], 53.9%–79.8%), 74.4% (95% CI, 56.3%–85.8%), and 84.3% (95% CI, 65.9%–93.2%) in the 45-mg, 30-mg, and 15-mg cohorts, respectively.

The rate of ≤0.1% *BCR::ABL1*^IS^ (MMR) by 5 years was highest with the 45-mg starting dose (46%) compared with the 30-mg (29%) and 15-mg (24%) cohorts; rates of ≤0.01% (MR4) and ≤0.0032% (MR4.5) *BCR::ABL1*^IS^ were similar between cohorts (Fig. 2B).

**Fig. 3:**
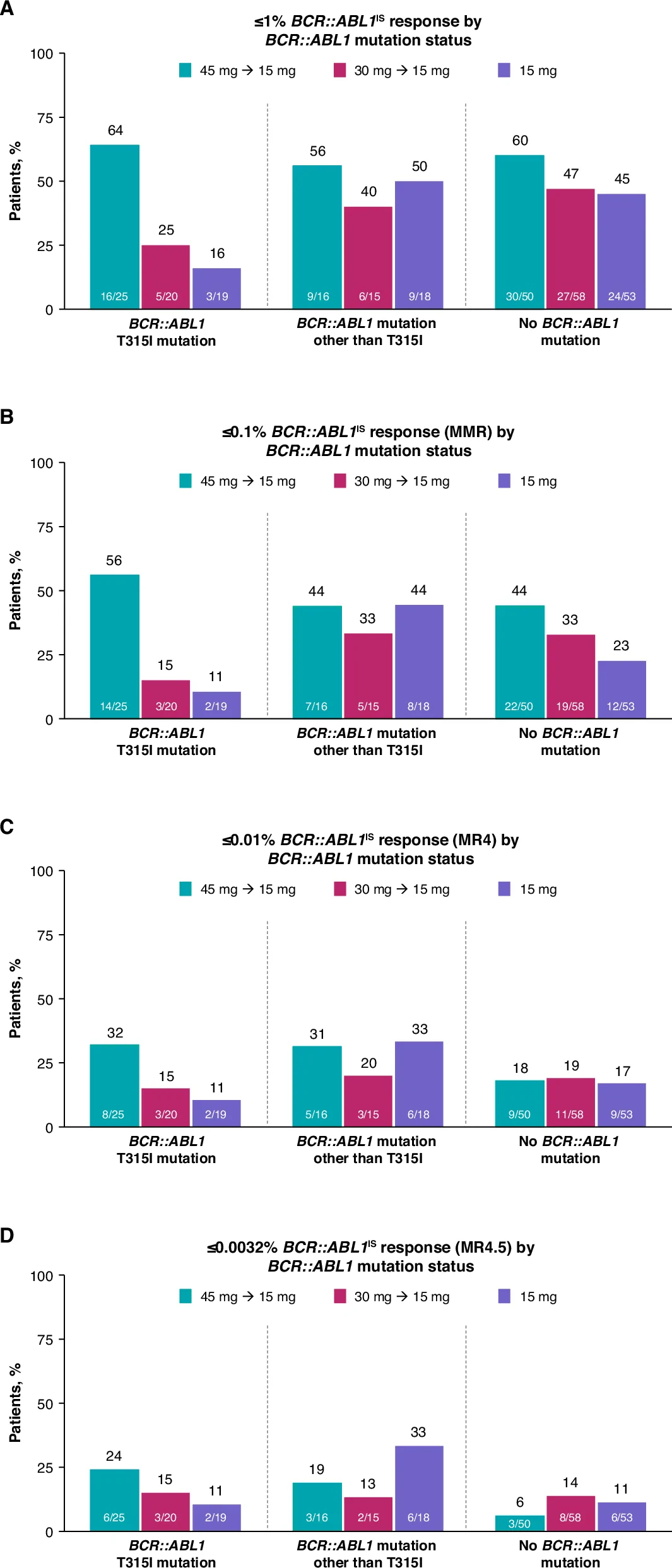
Response to ponatinib by baseline *BCR::ABL1* mutation status. Cumulative rates of **A** ≤1% *BCR::ABL1*^IS^ response, **B** MMR (≤0.1% *BCR::ABL1*^IS^), **C** MR4 (≤0.01% *BCR::ABL1*^IS^), and **D** MR4.5 (≤0.0032% *BCR::ABL1*^IS^) by 5 years according to baseline *BCR::ABL1* mutation status in the ITT population. ITT intention to treat, MMR major molecular response.

Regardless of baseline *BCR::ABL1* mutation status, patients achieved clinically meaningful *BCR::ABL1*^IS^ response rates, with the highest rates of ≤1% *BCR::ABL1*^IS^ response by 5 years in the 45-mg cohort. In patients with a T315I mutation, ≤1% *BCR::ABL1*^IS^ rates by 5 years were higher with the 45-mg starting dose (64%) compared with 30 mg (25%) and 15 mg (16%) (Fig. 3A); rates of ≤0.1% (MMR), ≤0.01% (MR4), and ≤0.0032% (MR4.5) *BCR::ABL1*^IS^ by 5 years were also higher in the 45-mg cohort compared with the 30-mg and 15-mg cohorts (Fig. 3B–D). In patients with non-T315I mutations or no mutations, ≤1% *BCR::ABL1*^IS^ response rates were slightly higher in the 45-mg cohort compared with the 30-mg and 15-mg cohorts; however, the numerical difference in the rates between these cohorts was greater in patients with a T315I mutation, and ≤0.1% (MMR), ≤0.01% (MR4), and ≤0.0032% (MR4.5) *BCR::ABL1*^IS^ response rates were generally similar between cohorts. Among the 10 patients with a T315I mutation in combination with ≥1 additional non-T315I mutation, 3 achieved ≤1% *BCR::ABL1*^IS^ by 5 years (45-mg cohort, *n* = 2; 15-mg cohort, *n* = 1), all of whom were on treatment for >5 years; 5 of the remaining 7 patients without ≤1% *BCR::ABL1*^IS^ response at 5 years were on treatment for approximately 1 year or more.

### Maintenance of response after dose reduction

Dose reduction to 15 mg after achieving ≤1% *BCR::ABL1*^IS^ occurred in 80% (45/56) of patients in the 45-mg cohort and 71% (27/38) of patients in the 30-mg cohort. In the remaining patients, dose modifications due to AEs were made per protocol and clinical judgement by the investigator. Among patients with dose reductions to 15 mg after achieving response, maintenance of ≤1% *BCR::ABL1*^IS^ was observed in 69% (31/45) of patients who started at 45 mg and 78% (21/27) of those who started at 30 mg, whereas responses were lost in 31% (14/45) and 22% (6/27), respectively. In patients who lost response following dose reduction to 15 mg, 86% (12/14) and 67% (4/6) who started at 45 mg and 30 mg, respectively, dose re-escalated their dose.

Among all patients who lost response regardless of when/if dose reduction occurred (before or after achieving ≤1% *BCR::ABL1*^IS^, or never) and reason for dose reduction (achievement of ≤1% *BCR::ABL1*^IS^ or AE occurrence), dose was re-escalated in 76% (13/17) in the 45-mg cohort and 56% (5/9) in the 30-mg cohort. Responses were regained after dose re-escalation in 69% (9/13) of patients with dose re-escalation in the 45-mg cohort and 80% (4/5) in the 30-mg cohort.

### Disease progression and survival

Progression to AP-CML occurred in 11 (12%), 11 (12%), and 13 (14%) patients in the 45-mg, 30-mg, and 15-mg cohorts, respectively, while on study. Progression to BP-CML occurred in 3 (3%), 1 (1%), and 2 (2%) patients, respectively, after study end.

**Fig. 4:**
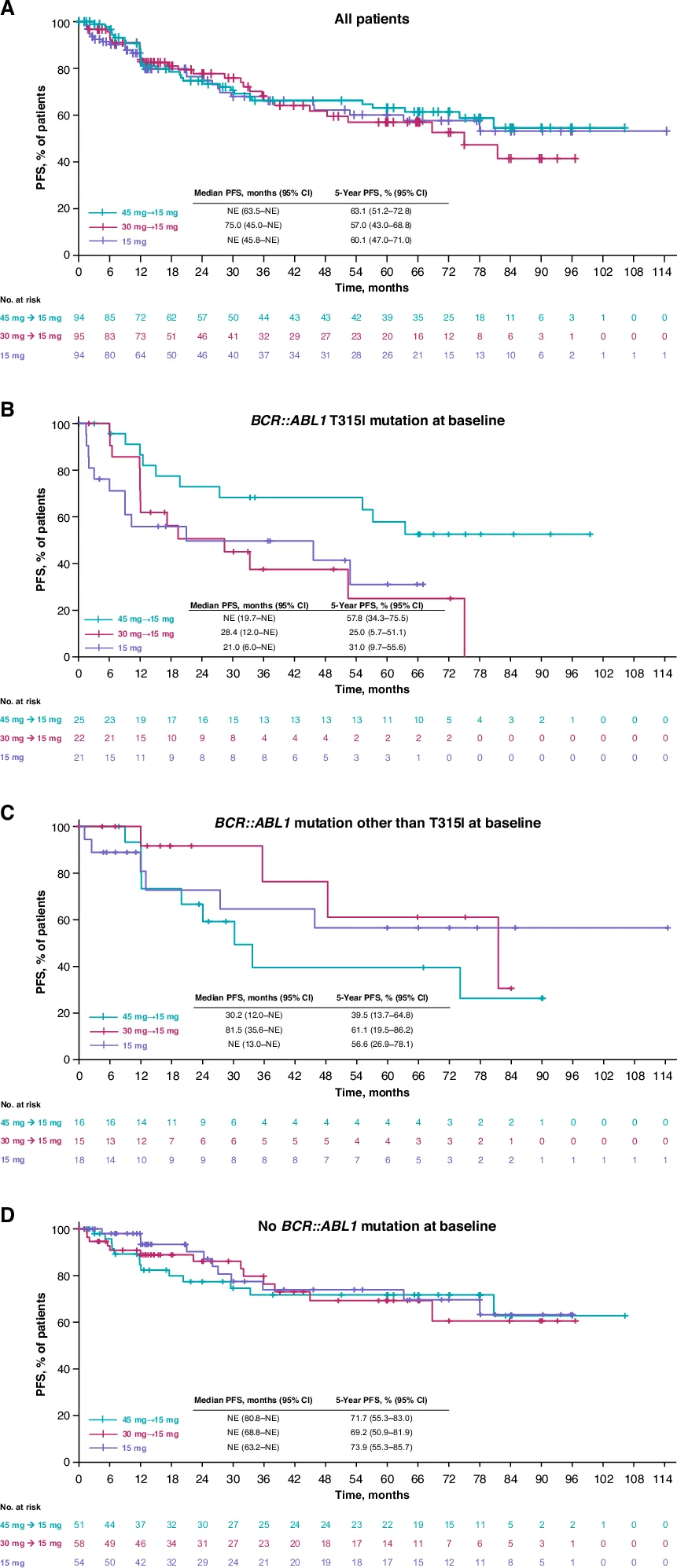
PFS in all patients and by baseline *BCR::ABL1* mutation status. **A** Overall population, **B** patients with a baseline *BCR::ABL1* T315I mutation, **C** patients with a *BCR::ABL1* mutation other than T315I, and **D** patients with no *BCR::ABL1* mutations. Survival probability was estimated using the Kaplan–Meier method in all randomized patients. Tick marks represent censored patients. CI confidence interval, NE not estimable, PFS progression-free survival.

Median PFS was not reached in the 45-mg or 15-mg cohorts and was 75.0 months in the 30-mg cohort; Kaplan–Meier–estimated 5-year PFS rates were 63%, 57%, and 60% in the 45-mg, 30-mg, and 15-mg cohorts, respectively (Fig. 4A). In patients with a *BCR::ABL1* T315I mutation at baseline, median PFS was not reached, 28.4 months, and 21.0 months in the 45-mg, 30-mg, and 15-mg cohorts, respectively; estimated PFS rates at 5 years were 58%, 25%, and 31%, respectively (Fig. 4B). In patients with non-T315I *BCR::ABL1* mutations at baseline, median PFS was 30.2 months, 81.5 months, and not reached in the 45-mg, 30-mg, and 15-mg cohorts, respectively; estimated PFS rates at 5 years were 40%, 61%, and 57%, respectively (Fig. 4C). In patients with no *BCR::ABL1* mutations at baseline, median PFS was not reached in the 45-mg, 30-mg, and 15-mg cohorts; estimated PFS rates at 5 years were 72%, 69%, and 74%, respectively (Fig. 4D).

**Fig. 5:**
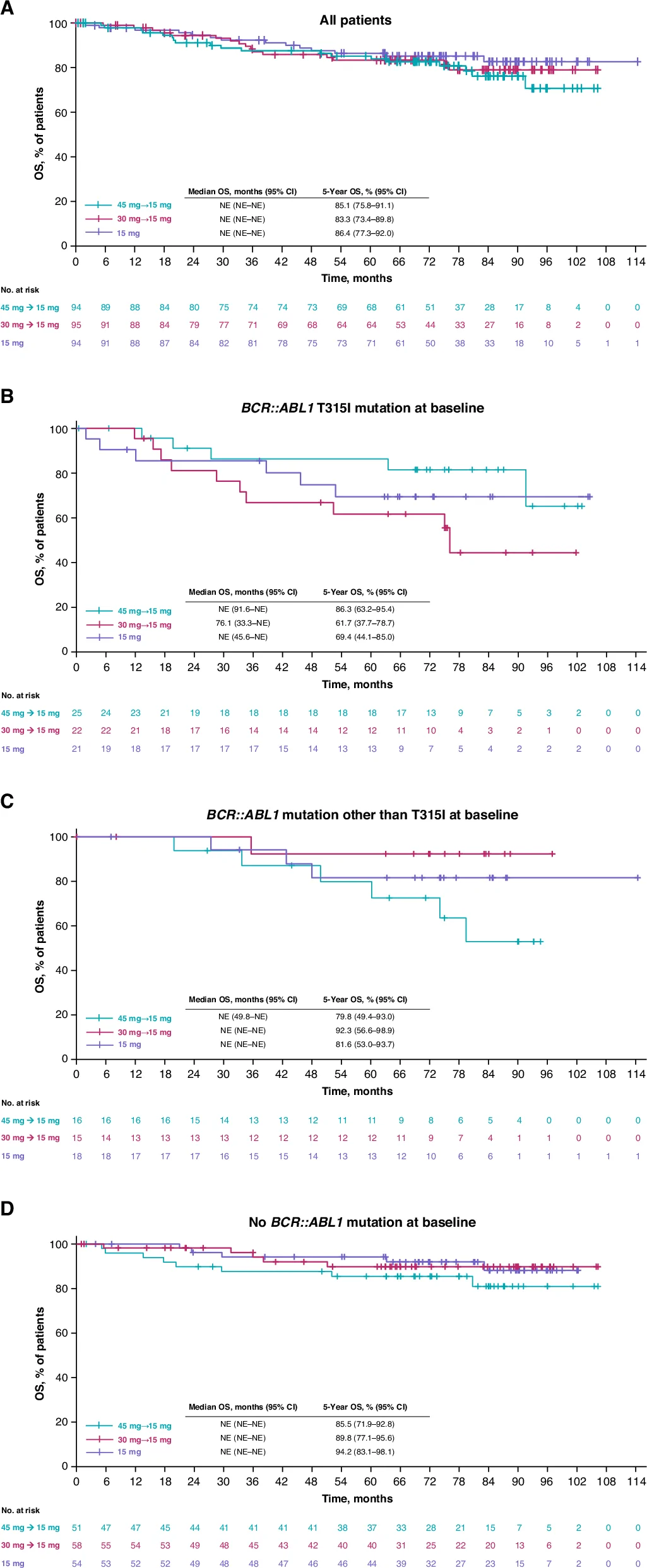
OS in all patients and by baseline *BCR::ABL1* mutation status. **A** Overall population, **B** patients with a baseline *BCR::ABL1* T315I mutation, **C** patients with a *BCR::ABL1* mutation other than T315I, and **D** patients with no *BCR::ABL1* mutations. Survival probability was estimated using the Kaplan–Meier method in all randomized patients. Tick marks represent censored patients. CI confidence interval, NE not estimable, OS overall survival.

Median OS was not reached, with Kaplan–Meier–estimated 5-year OS rates exceeding 80% in each cohort (Fig. 5A). Median OS was not reached in all 3 dosing cohorts regardless of baseline *BCR::ABL1* mutation status, except for patients with a T315I mutation in the 30-mg cohort (median OS, 76.1 months). In patients with a T315I mutation, estimated OS rates at 5 years were 86%, 62%, and 69%, in the 45-mg, 30-mg, and 15-mg cohorts, respectively (Fig. 5B). In patients with non-T315I mutations, estimated OS rates at 5 years were 80%, 92%, and 82%, respectively (Fig. 5C). In patients with no mutations, estimated OS rates at 5 years were 86%, 90%, and 94%, respectively (Fig. 5D).

### Safety and tolerability

Rates of TEAEs and grade ≥3 TEAEs were similar across cohorts (Table 1). In the safety population, the most common any-grade nonhematologic TEAEs were hypertension (35%; 33/98 had a history of hypertension [45-mg cohort, 13/31; 30-mg cohort, 13/39; 15-mg cohort, 7/28]), headache (22%), and alanine aminotransferase (ALT) increase (21%); the most common grade 3/4 nonhematologic TEAEs were hypertension (10%), lipase increase (7%), and ALT increase (4%). The most common hematologic TEAEs were thrombocytopenia (40%; grade 3/4: 27%), neutropenia (27%; grade 3/4: 18%), and anemia (21%; grade 3/4: 8%). Similar proportions of patients in the 45-mg, 30-mg, and 15-mg cohorts experienced any-cause serious TEAEs and treatment-related serious TEAEs; exposure-adjusted rates of serious TEAEs per 100 patient-years were 14.4 (95% CI: 8.9–19.8), 17.0 (95% CI: 10.5–23.5), and 17.7 (95% CI: 11.1–24.3) in the 45-mg, 30-mg, and 15-mg cohorts, respectively. Dose reductions and interruptions for TEAEs were more frequent in the 45-mg cohort compared with the 30-mg and 15-mg cohorts, but rates of treatment discontinuation due to TEAEs were comparable across cohorts (Table 1). There were 19 deaths (20%) in the 45-mg cohort, 16 (17%) in the 30-mg cohort, and 14 (15%) in the 15-mg cohort. The cause of death was related to disease progression in 7/19, 8/16, and 5/14 deaths, respectively. Other causes of death included stomach cancer, cardiorespiratory arrest, loss of consciousness, sudden death, pneumonia, infection, septic shock, digestive hemorrhage, and intracranial hemorrhage in the 45-mg cohort; pulmonary embolism, septic shock, cardiac arrest, transplant-related complications or infection, pancreatic cancer, and pulmonary infection in the 30-mg cohort; and COVID-19 pneumonia, pneumonia, and refractory chronic lymphocytic leukemia in the 15-mg cohort. The safety profile by baseline *BCR::ABL1* mutation status was generally similar to the overall safety population.

**Table 1:** Overview of TEAEs (safety population).

Event, *n* (%)	45 mg → 15 mg (*n* = 94)		30 mg → 15 mg (*n* = 94)		15 mg (*n* = 94)	
Any TEAE	94 (100)		92 (97.9)		92 (97.9)	
Treatment-related	91 (96.8)		86 (91.5)		82 (87.2)	
Grade 3/4 TEAE	66 (70.2)		62 (66.0)		64 (68.1)	
Treatment-related	60 (63.8)		53 (56.4)		57 (60.6)	
Serious TEAE	38 (40.4)		33 (35.1)		39 (41.5)	
Treatment-related	27 (28.7)		25 (26.6)		27 (28.7)	
Grade 5 TEAE^a^	4 (4.3)		2 (2.1)		3 (3.2)	
Discontinuations due to TEAE^b^	23 (24.5)		18 (19.1)		19 (20.2)	
Thrombocytopenia	3 (3.2)		5 (5.3)		4 (4.3)	
Acute coronary syndrome	1 (1.1)		2 (2.1)		0	
Hypertension	2 (2.1)		0		1 (1.1)	
Ischemic stroke	1 (1.1)		1 (1.1)		1 (1.1)	
Neutropenia	0		1 (1.1)		2 (2.1)	
Angina unstable	2 (2.1)		0		0	
Platelet count decreased	1 (1.1)		0		1 (1.1)	
Sudden death	2 (2.1)		0		0	
Dose reduction due to TEAE	48 (51.1)		37 (39.4)		32 (34.0)	
Dose interruption due to TEAE	76 (80.9)		64 (68.1)		62 (66.0)	
Deaths	19 (20.2)		16 (17.0)		14 (14.9)	
**Most common TEAEs**^**c**^	**All grades**	**Grade 3/4**	**All grades**	**Grade 3/4**	**All grades**	**Grade 3/4**
Hematologic TEAEs						
Thrombocytopenia	41 (43.6)	28 (29.8)	36 (38.3)	25 (26.6)	36 (38.3)	24 (25.5)
Neutropenia	29 (30.9)	17 (18.1)	23 (24.5)	18 (19.1)	25 (26.6)	15 (16.0)
Anemia	22 (23.4)	11 (11.7)	19 (20.2)	8 (8.5)	17 (18.1)	4 (4.3)
Platelet count decrease	11 (11.7)	5 (5.3)	9 (9.6)	6 (6.4)	11 (11.7)	8 (8.5)
Nonhematologic TEAEs						
Hypertension	31 (33.0)	10 (10.6)	39 (41.5)	12 (12.8)	28 (29.8)	6 (6.4)
ALT increase	26 (27.7)	4 (4.3)	9 (9.6)	1 (1.1)	23 (24.5)	6 (6.4)
Lipase increase	24 (25.5)	11 (11.7)	19 (20.2)	7 (7.4)	14 (14.9)	3 (3.2)
Headache	19 (20.2)	0	22 (23.4)	2 (2.1)	20 (21.3)	1 (1.1)
Pyrexia	17 (18.1)	1 (1.1)	7 (7.4)	2 (2.1)	10 (10.6)	0
Hypertriglyceridemia	16 (17.0)	3 (3.2)	10 (10.6)	1 (1.1)	9 (9.6)	1 (1.1)
AST increase	16 (17.0)	1 (1.1)	6 (6.4)	0	16 (17.0)	5 (5.3)
Constipation	14 (14.9)	0	11 (11.7)	0	14 (14.9)	0
Rash	13 (13.8)	0	9 (9.6)	0	4 (4.3)	0
Abdominal pain	12 (12.8)	1 (1.1)	11 (11.7)	1 (1.1)	11 (11.7)	2 (2.1)
Arthralgia	12 (12.8)	0	23 (24.5)	1 (1.1)	15 (16.0)	2 (2.1)
Dry skin	11 (11.7)	0	7 (7.4)	0	4 (4.3)	0
Fatigue	10 (10.6)	1 (1.1)	7 (7.4)	0	5 (5.3)	0
Back pain	8 (8.5)	0	15 (16.0)	1 (1.1)	8 (8.5)	1 (1.1)
Myalgia	8 (8.5)	1 (1.1)	9 (9.6)	1 (1.1)	12 (12.8)	0
Hyperglycemia	6 (6.4)	2 (2.1)	11 (11.7)	0	9 (9.6)	2 (2.1)

*ALT* alanine aminotransferase, *AST* aspartate aminotransferase, *TEAE* treatment-emergent adverse event.

^a^Number of deaths that occurred up to 30 days after the last ponatinib dose. In the 45-mg cohort, TEAEs with an outcome of death included cardiorespiratory arrest and myocardial ischemia in 1 patient, myocardial infarction and loss of consciousness in 1 patient, and sudden death in 2 patients. In the 30-mg cohort, TEAEs with an outcome of death included pulmonary embolism and cardiac arrest in 1 patient each. In the 15-mg cohort, TEAEs with an outcome of death included pneumonia in 2 patients and COVID-19 pneumonia in 1 patient.

^b^All TEAEs with “drug withdrawn” as the action taken; discontinuations in ≥2 patients (combined for the 3 cohorts).

^c^All-grade events in >10% of patients in any cohort; grade 3/4 events in >5% of patients in any cohort.

**Fig. 6:**
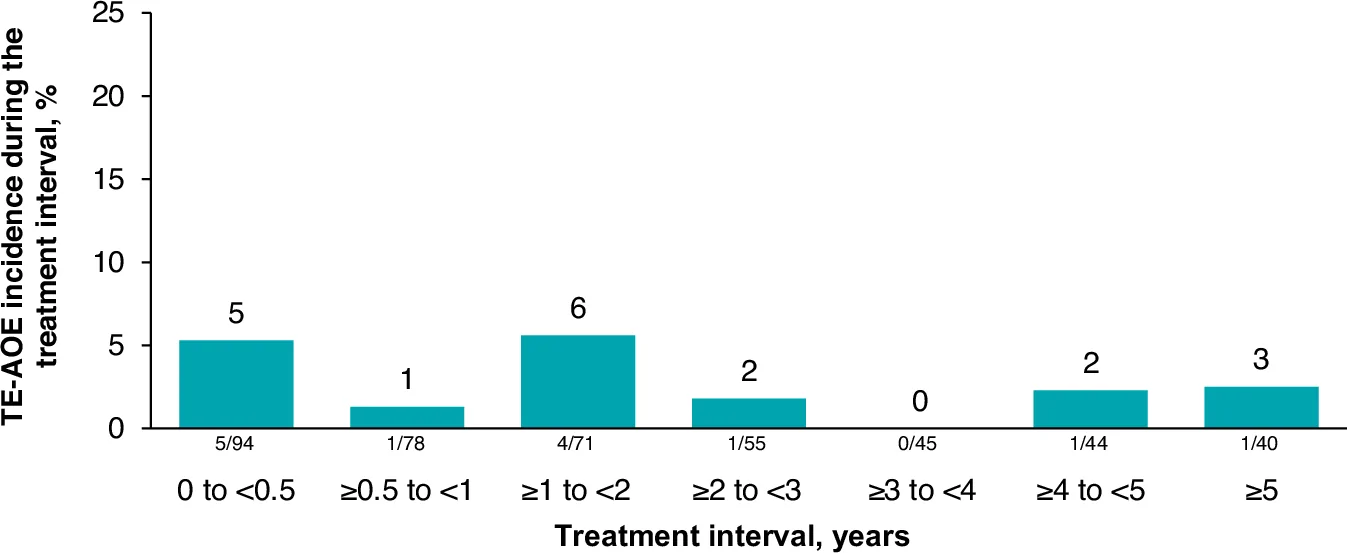
TE-AOE incidence by duration of ponatinib exposure in the 45-mg cohort. The 30-mg and 15-mg cohorts are not shown due to small sample sizes after year 3. TE-AOE treatment-emergent arterial occlusive event.

Twenty-seven patients (10%) had adjudicated treatment-emergent AOEs (TE-AOEs; Table 2). Exposure-adjusted rates of TE-AOEs per 100 patient-years were 4.1 in the 45-mg cohort, 3.8 in the 30-mg cohort, and 2.0 in the 15-mg cohort. Incidence of AOEs in the 45-mg cohort trended downward as the treatment period increased, with a low incidence (≤3%) after 2 years (Fig. 6). TE-AOEs occurred after protocol-mandated dose reduction upon achieving response in 9 patients (10%) in the 45-mg cohort and 4 patients (4%) in the 30-mg cohort; median time from dose reduction to first TE-AOE was 365 days (range, 11–1962) and 1152 days (range, 309–1494), respectively. Grade 3/4 TE-AOEs occurred in 19 patients (7%) overall and at similar rates between the 3 dosing cohorts. Eleven patients (4%) discontinued treatment because of TE-AOEs. Two patients in the 45-mg cohort had fatal AOEs (myocardial ischemia on study day 2075, myocardial infarction on study day 551); dose at AE onset was 15 mg and 30 mg, respectively. Baseline risk factors and characteristics of patients with TE-AOEs are shown in Supplementary Table S2. A low incidence of treatment-emergent venous thromboembolic events was observed, with 3 patients (1%; 1 in each cohort) experiencing an event. Incidence of TE-AOEs by baseline *BCR::ABL1* mutation status was generally similar to the overall safety population.

**Table 2:** Summary of TE-AOEs (safety population).

Event, *n* (%)	45 mg → 15 mg (*n* = 94)	30 mg → 15 mg (*n* = 94)	15 mg (*n* = 94)
Any TE-AOE	13 (13.8)	9 (9.6)	5 (5.3)
Serious TE-AOE	8 (8.5)	8 (8.5)	5 (5.3)
Grade 3/4 TE-AOE	6 (6.4)	8 (8.5)	5 (5.3)
TE-AOEs leading to any dose adjustment^a^	8 (8.5)	8 (8.5)	4 (4.3)
Discontinuation	5 (5.3)	4 (4.3)	2 (2.1)
Interruption	5 (5.3)	5 (5.3)	2 (2.1)
Reduction	0	2 (2.1)	0
Days on study at first observation of TE-AOE, median (range)	422 (13–2075)	563 (83–1627)	402 (381–2333)
Exposure-adjusted TE-AOEs, patients with events/100 person years (95% CI)^b^	4.1 (1.8–6.4)	3.8 (1.3–6.3)	2.0 (0.3–3.7)

*CI* confidence interval, *TE-AOE* treatment-emergent arterial occlusive event.

^a^TE-AOEs leading to treatment discontinuation, dose reduction, or dose interruption. A TE-AOE may be associated with more than one type of dose adjustment.

^b^For patients who experienced an event, the exposure contribution was the first dose date to event onset. For patients who did not experience an event, the exposure contribution was first dose date to last dose date.

### *BCR::ABL1* mutations at baseline and EOT

Baseline and EOT mutation data were available for 144 patients. To evaluate the effect of ponatinib in reducing pre-existing mutations, mutations were analyzed in patients with ≥1 mutation at baseline (*n* = 64) regardless of treatment discontinuation reason (including patients who transitioned off study to PTA). Of these patients, 18 had baseline mutations reduced below the level of detection (LOD), of which 8, 4, and 6 were in the 45-mg, 30-mg, and 15-mg cohorts, respectively (Supplementary Fig. S1A). Fourteen distinct mutations were no longer detected post-treatment (Supplementary Fig. S1B).

To evaluate the appearance of new mutations that might correlate with resistance, mutations were analyzed in 93 patients who discontinued ponatinib due to lack of efficacy or progressive disease. Among the 83 patients with available baseline and EOT mutation data, most did not acquire new mutations (*n* = 72, 86.7%; Supplementary Fig. S2). Among the remaining 11 patients with new detectable mutations, 5, 0, and 6 were from the 45-mg, 30-mg, and 15-mg cohorts, respectively; 6 had resulting T315I mutations in combination with another mutation at EOT (Supplementary Table S3).

## Discussion

The OPTIC trial was designed to define a response-based ponatinib dosing strategy capable of maintaining robust efficacy while improving long-term safety in patients with heavily pretreated CP-CML. With 5 years of follow-up, this final analysis confirms durable molecular response, sustained survival, and a manageable safety profile consistent with response-based dosing in third-line setting.

The 45-mg once daily starting dose, with reduction to 15 mg once daily upon achievement of ≤1% *BCR::ABL1*^IS^, demonstrated the highest rates of molecular response, particularly in patients with a baseline T315I mutation. These findings are clinically meaningful in a population with limited treatment options and historically inferior outcomes. Importantly, durable responses and favorable survival outcomes were observed across mutation categories, supporting ponatinib as an appropriate therapeutic option for patients requiring third-line therapy, including those with a T315I mutation.

The response-based dosing strategy allowed for early dose optimization while preserving efficacy. Maintenance of response following dose reduction and regained response following dose re-escalation support the clinical flexibility and adaptability of this approach, allowing clinicians to tailor therapy to patient response.

Deeper molecular responses were observed more frequently in patients who initiated ponatinib at 45 mg daily. Although OPTIC was not designed or powered for formal statistical comparisons between cohorts, the data suggest that a higher starting dose facilitates more rapid and deeper molecular responses, particularly in patients with T315I mutations. However, despite these differences in molecular response depth and kinetics, PFS and OS rates were generally similar across cohorts outside of the T315I subgroup, whereas TE-AOE rates were numerically lower in the 15-mg cohort. These findings suggest that, in heavily pretreated CP-CML, long-term clinical outcomes may depend not only on achieving deep molecular responses, but also on maintaining adequate disease control and sustained treatment exposure with manageable toxicity. In this context, achieving and maintaining ≤1% *BCR::ABL1*^IS^ may be sufficient to preserve long-term benefit for many patients [15–17], while deeper responses may primarily reflect enhanced disease suppression rather than a direct survival advantage. Together, these observations support a response-based dosing strategy with ponatinib that seeks to balance rapid disease control, long-term efficacy, and safety.

Over time, incidence of AOEs declined in patients who started ponatinib at 45 mg, particularly beyond 2 years of therapy, consistent with reduced cumulative exposure after dose reduction. The exposure-adjusted AOE rate with the 45-mg dose in OPTIC was nearly equivalent to that with the 30-mg dose (4.1 and 3.8 patients per 100 patient years, respectively) and approximately half the value in PACE (8.9 patients per 100 patient years) [18]. A prior analysis comparing outcomes in patients with CP-CML who received 45 mg/day of ponatinib in PACE or OPTIC showed that the risk for AOEs in OPTIC was ~60% lower than in PACE [19]. Importantly, however, the median age of the PACE population was substantially higher than that of the OPTIC population [18, 19], which may have contributed to differences in observed AOE rates and limits direct cross-trial comparisons. In addition, improved contemporary cardiovascular monitoring and management may also have influenced outcomes. AOE mitigation strategies, including smoking cessation and aggressive management of hypertension, hyperlipidemia, diabetes, and other cardiovascular risk factors, may be valuable for reducing cardiovascular risk during ponatinib treatment. Collectively, these findings support the ability of a response-based ponatinib dosing strategy to maintain clinical benefit while improving long-term tolerability.

Mutation analyses further support the durability of ponatinib benefit. Several pre-existing mutations were no longer detectable at EOT. These mutations were consistent with ponatinib’s selectivity profile from preclinical cellular studies [6]. This suggests that ponatinib may reduce clones containing select *BCR::ABL1* mutations below the LOD, contributing to reduced disease burden.

Most patients who discontinued treatment due to lack of efficacy or progressive disease did not develop new detectable mutations during therapy. Interestingly, a few patients with T315I mutations at baseline acquired a second non-T315I mutation at EOT, and 1 patient with non-T315I mutations at baseline acquired a T315I mutation at EOT. While our analyses could not determine whether these mutations were compound, preclinical data have shown that ponatinib reduced activity in certain compound T315I mutation combinations [6], suggesting that additional mutations may have emerged in the same T315I clone in this analysis. These findings indicate that leveraging ponatinib as early as indicated (i.e., after 2 prior TKIs), before patients acquire even a single mutation, may provide optimal clinical benefit. An analysis of patients from PACE and OPTIC who received a starting dose of 45 mg daily and had prior second-generation TKI exposure reported that the 2-year ≤1% *BCR::ABL1*^IS^ response rate was similar for patients receiving ponatinib after prior treatment with 1 or ≥2 second-generation TKIs, but 2-year PFS rates were higher in patients with 1 prior second-generation TKI [20]. Furthermore, incidence of AOEs was lower in OPTIC patients treated with ≤2 versus ≥3 prior second-generation TKIs [20]. These results further support initiating ponatinib as early as indicated.

Comparative analyses with other TKIs, including asciminib, should be interpreted cautiously in the absence of randomized head-to-head data. Indirect comparisons suggest favorable response rates with ponatinib vs second-generation TKIs in the third-line setting [21, 22]. In a propensity score matching analysis, 3-year PFS and OS rates were significantly greater with third-line ponatinib versus second-generation TKIs, respectively; ponatinib was an independent predictor of better survival in multivariate analysis [22]. In a matching-adjusted indirect comparison study, ponatinib was associated with higher response rates at 1 year than asciminib, including in patients with a T315I mutation [23]. AOE rates with asciminib were comparable to ponatinib 45 mg [11, 24, 25]. Notably, higher and more frequent doses are required for asciminib (200 mg twice daily) in patients with T315I mutations, which is substantially more expensive than the standard asciminib 40 mg twice daily dose or ponatinib 45 mg once daily [26–29]. Overall, these data support a potential greater benefit with ponatinib compared with asciminib and similar safety with optimized ponatinib dosing.

Limitations of this study include its open-label design, relatively small sample sizes in some analyses and mutation subgroups, and limited data in patients who discontinued treatment. Nevertheless, this 5-year follow-up provides one of the most comprehensive long-term datasets available in third-line CP-CML.

In conclusion, 5-year OPTIC results demonstrate that response-based ponatinib dosing (45 mg QD reduced to 15 mg QD) provides durable efficacy and sustained mutation control with a favorable benefit:risk profile in patients with TKI-resistant CP-CML. These findings reinforce ponatinib as an important option in the third-line setting, particularly for patients with T315I mutations, and emphasize the importance of selecting therapy based on individual molecular and clinical features.

## Supplementary information

Supplementary Information

## Data Availability

The data sets, including the redacted study protocol, redacted statistical analysis plan, and individual participant data supporting the results reported in this article, will be made available within 3 months from initial request to researchers who provide a methodologically sound proposal. The data will be provided after de-identification, in compliance with applicable privacy laws, data protection, and requirements for consent and anonymization.
