# Supplementary material for: Five-year follow-up of OPTIC: long-term efficacy, safety, and mutation analyses of ponatinib in chronic-phase chronic myeloid leukemia from a randomized phase 2 trial
Source: Leukemia. 2026 Jul 24;40(9):1882–92. doi: 10.1038/s41375-026-03009-x (PMC13506327; doi:10.1038/s41375-026-03009-x)
Supplement: Supplementary file 1 [file 41375_2026_3009_MOESM1_ESM.pdf]

Supplementary Information

Supplementary Tables

Table S1. Patient demographics and baseline disease characteristics.

Characteristic	45 mg → 15 mg (n = 94)	30 mg → 15 mg (n = 94)	15 mg (n = 94)
Median (range) age, y	46 (19–81)	51 (21–77)	49 (18–81)
Sex, male, n (%)	50 (53)	38 (40)	53 (56)
ECOG PS score 0 or 1, n (%)	93 (99)	93 (99)	94 (100)
Median (range) time since diagnosis, y	5.5 (1–21)	5.1 (1–29)	5.7 (1–22)
≥1 CV risk factor,[a] n (%)	32 (34)	30 (32)	32 (34)
Stopped prior TKI for resistance, n (%)	92 (98)	94 (100)	94 (100)
Best response to last prior TKI, n (%)			
CHR or worse	61 (65)	55 (59)	57 (61)
BCR::ABL1 mutation at baseline, n (%)[b]			
No mutation	51 (54)	58 (62)	54 (57)
Any mutation	41 (44)	35 (37)	39 (42)
T315I	25 (27)	21 (22)	21 (22)
Mutation other than T315I[c]	16 (17)	14 (15)	18 (19)
G250E	7 (7)	2 (2)	4 (4)
F317L	2 (2)	4 (4)	2 (2)
F359V	1 (1)	1 (1)	5 (5)
E255K	1 (1)	2 (2)	3 (3)
V299L	3 (3)	1 (1)	2 (2)
M244V	1 (1)	2 (2)	2 (2)
≥2 mutations detected	10 (11)	6 (6)	5 (5)
≥2 mutations with T315I mutation and another mutation(s)[d]	3 (3)	3 (3)	4 (4)

Abbreviations: CHR, complete hematologic response; CV, cardiovascular; ECOG PS, Eastern Cooperative Oncology Group performance status; TKI, tyrosine kinase inhibitor.

[a]CV risk factors included hypertension, diabetes, and hypercholesterolemia.

[b]Sanger sequencing was used for mutation testing. Four patients (45 mg, n = 2; 30 mg, n = 1; 15 mg, n = 1) did not have mutation testing performed at baseline.
[c]Mutations identified in at least 5 patients (combined for the 3 cohorts) are presented.
[d]Other mutations included V299L (1 patient) and G250E (2 patients) in the 45-mg cohort, F317L, M244V, and E255K (1 patient each) in the 30-mg cohort, and F359V, G250E, E255K, and M244V/L248V (1 patient each) in the 15-mg cohort.
Adapted from *Blood*, Volume 138, Cortes J, Apperley J, Lomaia E, et al. Ponatinib dose-ranging study in chronic-phase chronic myeloid leukemia: a randomized, open-label phase 2 clinical trial, 2042-2050, © 2021, with permission from The American Society of Hematology.

Table S2. Baseline risk factors and characteristics of patients with TE-AOEs.

Risk factor or characteristic, n	45 mg → 15 mg (n = 13)	30 mg → 15 mg (n = 9)	15-mg (n = 5)
Age			
18–49 years	5	1	2
50–64 years	5	5	2
65–74 years	2	3	1
≥75 years	1	0	0
Sex			
Male	4	3	2
Female	9	6	3
BCR::ABL1 mutation at baseline			
T315I mutation	2	3	1
Mutation other than T315I	6	3	0
No mutation	5	3	4
History of hypertension			
Yes	3	5	1
No	10	4	4
History of diabetes			
Yes	1	0	0
No	12	9	5
Obesity			
Yes	1	1	2
No	12	8	3
History of hypercholesterolemia			
Yes	2	2	1
No	11	7	4

Abbreviation: TE-AOE, treatment-emergent arterial occlusive event.

Table S3. New *BCR::ABL1* mutations identified at EOT in patients who discontinued treatment due to lack of efficacy or progressive disease (n = 93[a]).

Dose group	Mutation at baseline	Mutation at EOT	Treatment duration (months)
No mutations at baseline			
45 mg → 15 mg	None detected	E255K	75.3
15 mg	None detected	T315I	4.2
15 mg	None detected	T315I	18.9
15 mg	None detected	T315I	45.0
15 mg	None detected	L248V	75.3
Single T315I mutation at baseline			
45 mg → 15 mg	T315I	T315I/F359V	3.7
45 mg → 15 mg	T315I	T315I/Q252H	8.4
45 mg → 15 mg	T315I	T315I/E450G	16.6
15 mg	T315I	T315I/E255K	9.3
15 mg	T315I	T315I/E255K	25.0
Multiple mutations at baseline			
45 mg → 15 mg	F311V/L387F/H396R/E459K	T315I/H396R	13.9

Abbreviation: EOT, end of treatment.

Each row represents 1 patient.

[a]83 patients had paired baseline and EOT mutation data available.

Supplementary Figures

Supplementary Fig. S1. Suppression of baseline *BCR::ABL1* mutations below the LOD at EOT regardless of reason for EOT (including study termination). (A) Mutation status at baseline and EOT in patients who lost mutations and **(B)** location of eliminated mutations. **Abbreviations:** EOT, end of treatment; LOD, level of detection; PD, progressive disease.

A

45 mg → 15 mg

Mutations at baseline	Mutations at EOT	Treatment duration (months)	Reason for EOT
F317L/E459G	F317L	10.9	Lack of efficacy
G250E/F359V	F359V	5.8	Adverse event
E255V/ V299L	E255V	28.1	Lack of efficacy
F311V/L387F/ H396R/E459K	T315I/H396R	13.9	Lack of efficacy
G250E/V299L	L248R/G250E /Y253H	24.6	Adverse event
H396R	None detected	22.1	Adverse event
G250E/T315I	T315I	60.7	Lack of efficacy
E459K	None detected	23.2	Withdrawal by patient

30 mg → 15 mg

Mutations at baseline	Mutations at EOT	Treatment duration (months)	Reason for EOT
T315I/F317L	T315I	15.4	PD
M244V/T315I	T315I	31.9	Lack of efficacy
V379I/P441L	P441L	13.6	Lack of efficacy
E255K/T315I	T315I	11.0	Lack of efficacy

15 mg

Mutations at baseline	Mutations at EOT	Treatment duration (months)	Reason for EOT
T315I	None detected	33.3	Lack of efficacy
F317L	None detected	54.1	Lack of efficacy
M244V/L248V /T315I	T315I	8.4	PD
Y253H/F359V	Y253H	76.4	Study terminated by sponsor
V299L	None detected	3.6	Adverse event
E255K/T315I	E255K	2.8	Lack of efficacy

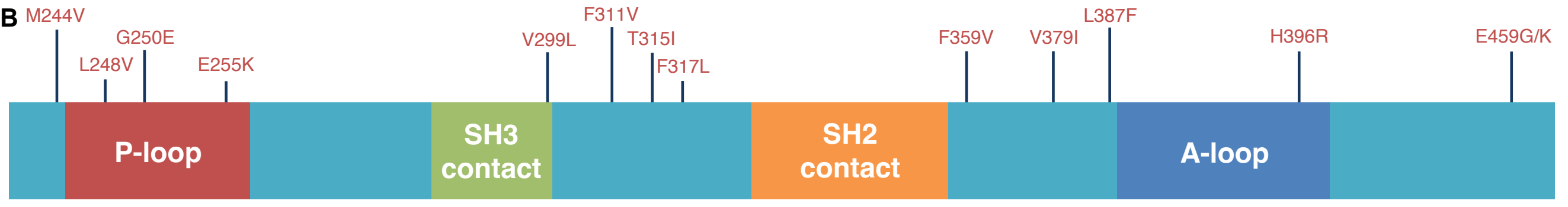
B
M244V
G250E
L248V
E255K
V299L
F311V
T315I
F317L
F359V
V379I
L387F
H396R
E459G/K
P-loop
SH3 contact
SH2 contact
A-loop

Supplementary Fig. S2. Flowchart of baseline and EOT *BCR::ABL1* mutations in patients who discontinued treatment due to lack of efficacy or progressive disease. Row 2 of the flowchart represents all patients who discontinued treatment due to lack of efficacy or progressive disease by baseline mutation status, and row 3 represents those patients who also had paired EOT mutation data available. EOT mutation status (no new mutation, new mutation, or loss of mutation) in each group with paired baseline and EOT data available is shown in row 4. **Abbreviation:** EOT, end of treatment.

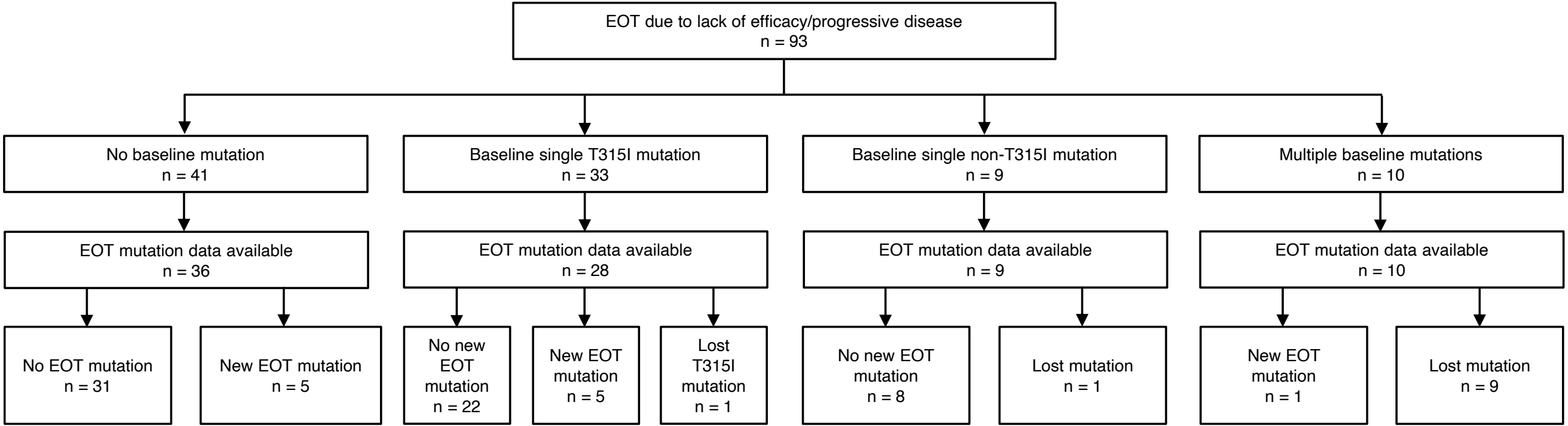
EOT due to lack of efficacy/progressive disease
n = 93
No baseline mutation
n = 41
Baseline single T315I mutation
n = 33
Baseline single non-T315I mutation
n = 9
Multiple baseline mutations
n = 10
EOT mutation data available
n = 36
EOT mutation data available
n = 28
EOT mutation data available
n = 9
EOT mutation data available
n = 10
No EOT mutation
n = 31
New EOT mutation
n = 5
No new EOT mutation
n = 22
New EOT mutation
n = 5
Lost T315I mutation
n = 1
No new EOT mutation
n = 8
Lost mutation
n = 1
New EOT mutation
n = 1
Lost mutation
n = 9
